# A Role for the Claustrum in Salience Processing?

**DOI:** 10.3389/fnana.2019.00064

**Published:** 2019-06-19

**Authors:** Jared B. Smith, Glenn D. R. Watson, Zhifeng Liang, Yikang Liu, Nanyin Zhang, Kevin D. Alloway

**Affiliations:** ^1^Molecular Neurobiology Laboratory, Salk Institute for Biological Studies, La Jolla, CA, United States; ^2^Department of Psychology and Neuroscience, Duke University, Durham, NC, United States; ^3^Laboratory for Comparative Neuroimaging, Institute for Neuroscience, Chinese Academy of Sciences, Shanghai, China; ^4^Center for Neural Engineering, Penn State University, Millennium Science Complex, University Park, PA, United States; ^5^Department of Biomedical Engineering, Penn State University, Millennium Science Complex, University Park, PA, United States; ^6^Huck Institute of Life Sciences, Penn State University, Millennium Science Complex, University Park, PA, United States; ^7^Neural and Behavioral Sciences, Center for Neural Engineering, Pennsylvania State University, University Park, PA, United States

**Keywords:** claustrum, amygdala, insula, salience network, functional connectivity, anatomical connectivity, medial prefrontal cortex, thalamus

## Abstract

The claustrum (CLA) is a subcortical structure, present only in mammals, whose function remains uncertain. Previously, using resting-state functional magnetic resonance imaging (rs-fMRI) in awake head-fixed rats, we found evidence that the CLA is part of the rodent homolog of the default mode network (DMN; Smith et al., 2017). This network emerged as strong functional connections between the medial prefrontal cortex (mPFC), mediodorsal (MD) thalamus, and CLA in the awake state, which was not present following administration of isoflurane anesthesia. In the present report, we review evidence indicating that the rodent CLA also has connections with structures identified in the rodent homolog of the salience network (SN), a circuit that directs attention towards the most relevant stimuli among a multitude of sensory inputs (Seeley et al., 2007; Menon and Uddin, 2010). In humans, this circuit consists of functional connections between the anterior cingulate cortex (ACC) and a region that encompasses both the CLA and insular cortex. We further go on to review the similarities and differences between the functional and anatomical connections of the CLA and insula in rodents using both rs-fMRI and neuroanatomical tracing, respectively. We analyze in detail the connectivity of the CLA with the cingulate cortex, which is a major node in the SN and has been shown to modulate attention. When considered with other recent behavior and physiology studies, the data reveal a role for the CLA in salience-guided orienting. More specifically, we hypothesize that limbic information from mPFC, MD thalamus, and the basolateral amygdala (BLA) are integrated by the CLA to guide modality-related regions of motor and sensory cortex in directing attention towards relevant (i.e., salient) sensory events.

## Introduction

The claustrum (CLA) is a subcortical structure whose precise function remains unknown, but has been implicated in various mechanisms involved in directing attention (Mathur, 2014; Goll et al., 2015; Atlan et al., 2018; White et al., 2018), salience detection (Smythies et al., 2012), multisensory integration (Edelstein and Denaro, 2004), cross-modal transfer (Hadjikhani and Roland, 1998), perceptual binding (Crick and Koch, 2005), cognition (Jackson et al., 2018), and consciousness (Koubeissi et al., 2014; Stiefel et al., 2014; Kurada et al., 2019). In support of this view, the rodent CLA has extensive interhemispheric connections linking modality-related regions of sensory and motor cortex that control exploratory behaviors, such as visual cortex and the frontal eye fields that control visual search (Smith and Alloway, 2010; Smith et al., 2012, 2018; Alloway et al., 2014).

We recently used neuroanatomical tracing in conjunction with resting-state functional magnetic resonance imaging (rs-fMRI) in awake, head-fixed rats to investigate the relationship between the structural and functional connections of the rodent CLA (Smith et al., 2017). This study revealed connections from the CLA to several nodes in the default mode network (DMN) and in the salience network (SN); both of which are thought to serve distinct cognitive functions (Menon, 2011; Smith et al., 2017). In the awake state, our study revealed strong functional connections of the CLA with the medial prefrontal cortex (mPFC) and mediodorsal (MD) thalamus (i.e., nodes of the DMN) that are significantly attenuated in response to anesthetic induced loss of consciousness. In addition to the DMN, we observed strong functional connections of the CLA with cingulate cortex (Cg), a primary node in the human SN.

To gain further insights about the composition and function of the rodent homologs of the DMN and SN (Sforazzini et al., 2014; Gozzi and Schwarz, 2016), this article will examine the anatomical and functional connectivity between the CLA with nodes in both the DMN and SN. Based on recent behavioral and physiology findings on the CLA (Remedios et al., 2014; Atlan et al., 2018; Jackson et al., 2018; White et al., 2018), we propose that the CLA has a role in both salience-guided orienting and in the context-dependent regulation of an organism’s state of vigilance (e.g., varying degrees of anxiety related to threat). In support of this theory, anatomical studies have revealed strong inputs to the CLA from limbic structures, especially the basolateral amygdala (BLA; Atlan et al., 2018; Zingg et al., 2018). We hypothesize that these limbic inputs may provide “salience signals” to the CLA, which are then relayed to sensory and motor cortices to coordinate sensory exploration towards relevant stimuli. Our theory outlines clear proposals about future directions in CLA research to elucidate its role in emotional processing related to salience-guided sensory exploration.

### Intrinsic Connectivity Networks in the Human Brain

**Figure 1:**
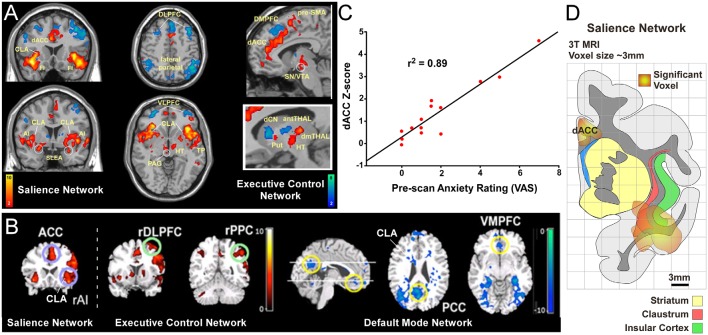
Human functional magnetic resonanceimaging (fMRI) studies have identified three distinct intrinsic connectivity networks (ICNs): salience, executive control, and default mode network (DMN). **(A)** Original human fMRI data identifying the salience network (SN; in hot colors) and the executive control network (ECN; in cool colors). The human SN primarily consists of the dorsal anterior cingulate cortex (dACC), anterior insula (AI), and fronto-insular cortex (FI; Seeley et al., 2007). **(B)** Graph from the same study showing a strong correlation between a pre-scan diagnostic of the subjects’ anxiety and the strength of connection within the SN. **(C)** Data from a separate study showing activity in the salience and ECNs (activation strength shown in hot colors), and also the DMN (deactivation strength shown in blue) in response to an auditory event transition. The human DMN primarily consists of connections between ventromedial prefrontal cortex (vmPFC) and posterior cingulate cortex (PCC) that appear as negative correlations when the salience and ECNs are active. **(D)** Reconstruction of SN activation area from upper left coronal section in panel **(A)** relative to anatomical boundaries for insula, claustrum and striatum (putamen, caudate, and accumbens). Note the “hotspot” in the insula for the SN encompasses the anatomical region containing the ventral portion of the claustrum (CLA). Panels **(A,C)** modified with permission from Seeley et al. (2007). Panel **(B)** modified with permission from Sridharan et al. (2008). Copyright (2008) National Academy of Science, USA.

The human brain is thought to be organized into interconnected regions that are functionally co-activated or co-deactivated during specific cognitive activities. The different regions in these large-scale brain networks show significant correlated blood oxygen-level dependent (BOLD) activity related to each function, as measured by fMRI (see Figure 1 and for a comprehensive review, see Menon, 2011). One such intrinsic connectivity network (ICN) is the DMN, which is thought to be active in the absence of all other overt and covert sensory, motor, cognitive, and emotional processing (Fox et al., 2005). The DMN consists of strongly correlated BOLD signals in the ventromedial prefrontal cortex (vmPFC), posterior cingulate cortex (PCC), and MD thalamus (Fox et al., 2005; Sridharan et al., 2008; Menon and Uddin, 2010).

Two more ICNs are thought to operate in opposition to the DMN: the SN and the executive control network (ECN; Figure 1; Seeley et al., 2007; Menon and Uddin, 2010; but see Murphy et al., 2009). In contrast to the DMN, the human SN (Figures 1A,B,D) is a distributed set of cortical and subcortical regions involved in detecting and responding to highly relevant (salient) stimuli (Seeley et al., 2007; Menon, 2011). The SN was first identified using conventional fMRI to measure BOLD responses during a spatial working memory task in human patients (Seeley et al., 2007). In this task, several cortical regions displayed significant BOLD responses, including the frontoinsular cortex. This region of insular cortex was subsequently used as a “seed region” for an independent component analysis of its functional connectivity patterns, some of which are shown in Figure 1A. The frontoinsular seed revealed strong functional connectivity to the dACC, as well as subcortical areas including MD thalamus, periaqueductal gray, extended amygdala, and others (Figure 1A). The known functions and modalities processed in these brain regions (including attention, sensory, visceral, affective, limbic, etc.) led the authors to conclude that this network, anchored by frontoinsular cortex and dACC, processes perceptual salience and thus termed it the “salience network.”

The discovery of the SN also revealed an interesting clinical relationship. Specifically, the strength of functional connectivity within the SN was strongly correlated (*r*^2^ = 0.89) with visual analog scores (VAS) from a pre-scan anxiety assay (Figure 1C). This correlation suggests a tight link between the strength of the SN and states of vigilance. Subsequent studies have replicated these findings and shown that functional connectivity within the SN is aberrant in many neurological disorders including anxiety (Geng et al., 2016), post-traumatic stress disorder (Brown et al., 2014), depression (Menon, 2011), psychosis with auditory delusions (Mallikarjun et al., 2018), and affective disorders (Menon and Uddin, 2010). As discussed below, this clinical finding has relevance in guiding experimental analysis of the SN in rodent models.

Owing to constraints in the spatial resolution of fMRI, it is difficult to distinguish the contribution of the insula from its surrounding brain regions, particularly the CLA. Conventional neuroanatomical tracing in non-human primates suggests that fMRI signals attributed to the anterior insula (AI) may arise, at least partly, from the CLA (Reser et al., 2016). In support of this view, overlays of SN activation regions onto an anatomical reconstruction show substantial involvement of the ventral CLA (Figure 1D). Closer inspection of this data indicates that the CLA is incorporated into both the SN and DMN in humans, with a stronger association to the SN (see Figures 1A,B). However, future human fMRI studies should directly test the relative degree to which the CLA is functionally involved in the SN or DMN. Additionally, although recent studies have examined the anatomical connectivity of the human CLA using diffusion tensor imaging (Milardi et al., 2013; Torgerson and Van Horn, 2014; Torgerson et al., 2015), more work is needed to assess the connectivity differences of the CLA with the adjacent insula. Together, such studies would enhance our understanding as to the functional and anatomical relationship between the insula and CLA with the DMN and SN nodes in the human brain.

### Claustrum Role in Rodent Homolog of Default Mode and Salience Networks

Large-scale ICNs have been thoroughly characterized in human and non-human primates, but less is known about their function in rodents (for comprehensive review, see Gozzi and Schwarz, 2016). Previous studies have attempted to delineate the rodent DMN and SN by combining anatomical tracing with rs-fMRI in anesthetized animals (Jonckers et al., 2011; Sforazzini et al., 2014). By contrast, our rs-fMRI imaging studies were performed in awake, head-fixed rats that were subsequently anesthetized with isoflurane, allowing us to examine the connectivity differences in the awake and anesthetized states (Zhang et al., 2010; Liang et al., 2011, 2012a, 2013, 2014, 2015; Smith et al., 2017). In both human and rodent studies, the DMN is sensitive to anesthesia, with strong attenuation of functional connectivity in this network in the anesthetized state compared to the awake state (Deshpande et al., 2010; Liu et al., 2015; Bukhari et al., 2017). In fact, this effect of anesthesia represented a major limitation in the interpretation of results reported by rodent imaging studies that used different anesthetic paradigms. By directly imaging BOLD signals during the awake and anesthetized states of the same animal, our work reveals how anesthesia alters the connectivity that is present in the awake, quiescent state.

**Figure 2:**
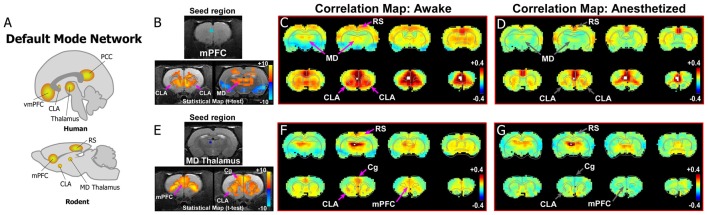
Dynamic functional connectivity in the rodent homolog of the DMN. **(A)** Diagrams illustrating the human and rodent DMNs. **(B–D)** Analysis of resting-state fMRI (rs-fMRI) in awake and anesthetized rats for a medial prefrontal cortex (mPFC) seed shows the rodent homolog of the DMN in the awake state. **(B)** Top panel shows location of seed region in mPFC in a structural T1-MRI. Bottom panels show statistical maps comparing correlation strength in the awake and anesthetized states (voxelwise two-way *t*-test), indicating a significant reduction in the mPFC functional connectivity with CLA and mediodorsal (MD) thalamus. Hot colors show *t*-values for voxels with *p* < 0.05 (false discovery rate), with correlations that significantly decrease in the anesthetized state compared to awake. **(C)** Correlation maps from rs-fMRI with seed-based correlation analysis of mPFC in the awake state reveal strong connections to CLA, MD thalamus and retrosplenial cortex (RS). Color plot indicates scale of correlation strength: hot colors are correlated, cool colors are anti-correlated. **(D)** Correlation maps from rs-fMRI with seed-based analysis of mPFC in the anesthetized state show weaker functional connections with CLA and MD thalamus. **(E–G)** Similar analyses of an MD thalamus seed show strong functional connections with mPFC and CLA in the awake state that are significantly attenuated under isoflurane-induced loss of consciousness. Data modified with permission from Smith et al. (2017).

As shown in Figure 2, data from our previous study reveals evidence for the rodent homolog of the DMN. The primary node for identifying the DMN from rs-fMRI seed-based analyses in humans is the vmPFC, which has strong functional connections with PCC (Figures 1, 2A). We, therefore, performed a similar rs-fMRI seed-based analysis of the homologous cortical area in rodents by using the prelimbic (PrL) cortex subdivision of mPFC as the seed region (Figures 2B–D; see also top row of Supplementary Figure S1). In the awake condition (Figure 2C), we observed strong functional connections between mPFC and retrosplenial cortex (RS), which represents the rodent homolog of PCC. Additionally, we observed strong functional mPFC connections with the CLA and MD thalamus. These connections were not present in the isoflurane-induced anesthetized state (Figure 2D), as indicated by significant reductions in the connectivity strength of the voxels in these regions (bottom panel Figure 2B). The same results were observed using an MD thalamus seed, which showed functional connections with the CLA and mPFC in the awake state that were subsequently lost in the anesthetized condition (Figures 2E–G; see also bottom row of Supplementary Figure S1). These results provide evidence of an mPFC-CLA-MD thalamus circuit, the rodent homolog of the DMN, which is disrupted by isoflurane anesthesia. Future studies with other anesthetic agents and/or mechanisms that regulate or disrupt consciousness, as well as electrophysiology studies, under the same conditions would be useful for confirming the contribution of the CLA in regulating the DMN.

**Figure 3:**
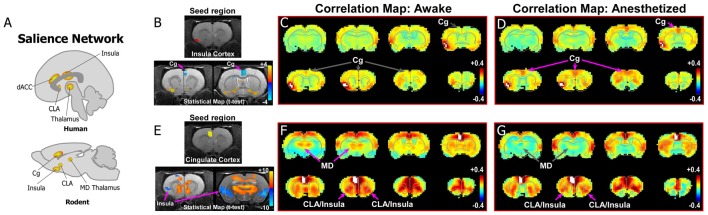
Rodent homolog of the SN. **(A)** Diagrams illustrating the human and rodent SN. **(B–D)** Analysis of rs-fMRI in awake and anesthetized rats for an insular cortex seed. **(B)** Top panel shows location of insular cortex seed in a structural T1-MRI. Bottom panel shows statistical maps comparing the awake and anesthetized states (voxelwise two-way *t*-test), indicating an increase in correlation strength between insula and cingulate cortex (Cg) in the anesthetized state compared to the awake state (blue area identified by pink arrow). Color plot shows *t*-values for voxels with *p* < 0.05 (false discovery rate), in which cool colors indicate a significant increase in correlation under anesthesia compared to the awake state. **(C)** Correlation maps from rs-fMRI with seed-based correlation analysis of insula in the awake state reveal no functional connections with Cg cortex. Color plot indicates scale of correlation strength: hot colors are correlated, cool colors are anti-correlated. **(D)** Correlation maps from rs-fMRI with seed-based analysis of insula in the isoflurane anesthetized state show weak functional connections with Cg cortex. **(E–G)** Similar analyses of a Cg cortex seed show strong functional connections with CLA in the awake and anesthetized states. Cg cortex also showed strong functional connections to MD thalamus in the awake state that is significantly diminished in the anesthetized state, similar to mPFC shown in Figure 2. Data modified with permission from Smith et al. (2017).

Similar to the DMN, recent studies have investigated the rodent homolog of the SN (for comprehensive review, see Gozzi and Schwarz, 2016). In the original report on the human SN, Seeley et al. (2007) define the circuit as anchored by frontoinsular cortex and dACC (Figures 1A,D, 3A). The human frontoinsular region, represented by Brodmann Areas 13 and 14, corresponds to regions of insular cortex in rodents located rostral to Bregma, but posterior to orbitofrontal cortex. The human dACC, which is represented by Brodmann Areas 24, 32, and 33, corresponds to Cg (Area 24) and PrL (Area 32) cortical areas in rodents. Therefore, to determine the nature of CLA connectivity with the rodent homolog of the SN network, we focused our analysis on its connectivity with the Cg, PrL, and insular cortical areas.

As the human SN was first identified by activity in the frontoinsula and its functional connections with dACC (Figures 1, 3A), we initially analyzed an insular seed in our rodent rs-fMRI data. Surprisingly, as seen in Figures 3B–D (see also top row of Supplementary Figure S2), an insular cortex seed analysis (seed region shown in Figure 3B) shows a weak connection between the insula and Cg cortex only under isoflurane anesthesia (Figure 3D). In contrast, shown in Figures 3E–G (see also bottom row of Supplementary Figure S2), a Cg cortex seed shows a strong connection specifically with the CLA in the awake state, but not insular cortex. This result is consistent with a recent rodent rs-fMRI study performed in a stronger magnetic field (7T), which reported that the CLA, but not insula, has functional connections with Cg cortex (Krimmel et al., 2019a).

When considered with the other recent literature on rodent rs-fMRI and homologs of human ICNs (Gozzi and Schwarz, 2016), our data support the presence of the SN and DMN in rat. More importantly, our results suggest that a more careful analysis in human/primate fMRI studies should assess the degree to which the insular cortex is involved in the SN, or if it should be re-defined as a Cg-CLA circuit only. It is possible that the insula is recruited into the SN only under certain conditions, particularly those related to known insular functions (e.g., interoceptive, visceral, etc; see Stephani et al., 2011). Our results also demonstrate unexpected dynamic changes in the functional connections of the CLA in the awake and anesthetized states that should be considered in future studies.

### Specificity of Functional Connectivity From Claustrum and Insula With rs-fMRI

Technical limitations and the geometric relationship of the CLA and insular cortex introduce problems with interpreting neuroimaging data related to these structures. While the CLA and insular cortex are separate structures in humans (Stephani et al., 2011), their close proximity often causes both regions to be included together in the large voxels used in human fMRI studies (Figure 1D). Thus, it is difficult to disambiguate the precise relationship between the BOLD signal and the activity of the actual neural substrate, as it pertains to the CLA or insula specifically (Reser et al., 2016).

**Figure 4:**
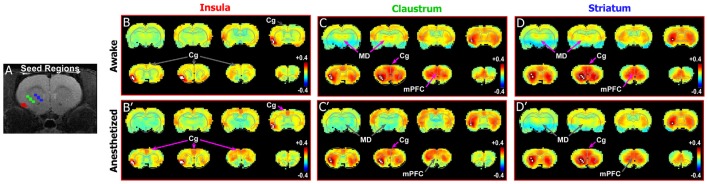
Differences in functional connectivity between striatum, claustrum, and insula. **(A)** Seed regions for insula (red), CLA (green), and striatum (blue) shown on T1-MRI. **(B–D′)** Correlation maps for insula, CLA, and striatum in awake state (top panels) and anesthetized states (bottom panels). Striatum and CLA had highly similar patterns of connectivity with strong connections to Cg cortex and MD thalamus in the awake state, whereas insula was only functionally connected with Cg cortexin the anesthetized state. Data modified with permission from Smith et al. (2017).

To test this relationship in rats, we compared the connectivity pattern produced by placing a seed in the granular and supragranular layers of insular cortex with the pattern revealed by placing a seed in a deeper area centered on the CLA. We then compared these patterns with the connectivity revealed by a seed in the striatum. As shown previously (Figure 3, Supplementary Figure S2) the Cg cortex had strong connectivity with the CLA, but not with the insula, in the awake state. This is further confirmed by seed analyses of the insula (Figures 4B,B′) and CLA (Figures 4C,C′). By comparison, the striatal seed revealed connectivity that was highly similar to the connectivity that emerged when a seed was placed in the CLA (Figures 4D,D′). This similarity is best explained by the fact that the CLA and the dorsal striatum have similar anatomical connections with the mPFC and Cg cortex (Smith and Alloway, 2014; Smith et al., 2016).

Because of the close proximity between the CLA and the surrounding insula, results produced by attempts at dissociating the functional connectivity between these two structures, even with higher magnet strengths in rodent studies (4.7–7T), must be interpreted cautiously (see new method by Krimmel et al., 2019a). We attempted to isolate the CLA from the insula in our study through seed-based analysis in an fMRI with a stronger field strength that allows for smaller voxel size. However, this can still be problematic because some evidence suggests that the CLA may be embedded in layer 6 of insular cortex (Mathur et al., 2009; Mathur, 2014). In support of our findings, a recent study using stronger magnetic fields (7T) for rs-MRI in humans found strong functional connections between the CLA and cingulate cortex, though they did not interrogate differences between claustrum and insula connections (Krimmel et al., 2019b). Thus, more work is needed to precisely clarify the structural and functional connectional differences between CLA and insula, particularly in animal models with a well delineated CLA, such as non-human primates. Such studies would allow for high-field rs-fMRI analyses with subsequent neuroanatomical tracing to make conclusive relationships between structural and functional data. Such studies would also be important for assessing any species differences in connections between these regions.

### Clarifying the Structural Connectivity of CLA and Insula With Neuroanatomical Tracing

In our previous anatomical tracing studies, combined injections of both retrograde and anterograde tracers into the CLA revealed substantial reciprocal connectivity with both mPFC and MD thalamus (Smith and Alloway, 2010, 2014; Smith et al., 2017). Interpreting the labeling from tracer injections in the CLA is difficult, however, because it requires tracer injections that are restricted to the CLA without infiltrating the surrounding insula and striatum.

**Figure 5:**
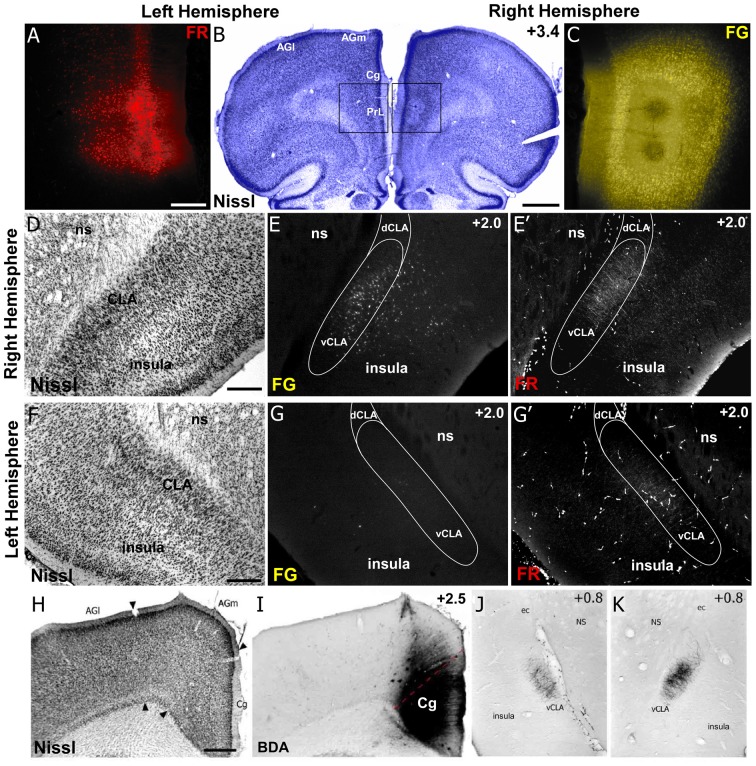
Neuroanatomical tracing clarifies the structural connectivity between mPFC, Cg, CLA, and insula. **(A–C)** Anterograde tracer injection (Fluororuby, FR) in the left hemisphere, shown in panel **(A)**, and retrograde tracer injection (Fluorogold, FG) in the right hemisphere, shown in panel **(C)**, into prelimbic cortex (PrL) of a rat. Injection site locations shown in Nissl stained coronal section in panel **(B)**. **(D–E′)** In the right hemisphere, retrograde labeling **(E)** is visible in both CLA and insular cortex, whereas anterograde labeling **(E′)** was found only in CLA. Panel **(D)** shows location of CLA in Nissl stained coronal section. **(F–G′)** Labeling in the left hemisphere shows very little retrograde labeling in insula or CLA **(G)**, but some anterograde labeling in the claustrum **(G′)**. **(H–K)** Injection of anterograde tracer BDA in Cg cortex shows bilateral labeling in claustrum but no labeling in insula. Numbers in upper right corner of panels indicate distance from Bregma in millimeter. Scale bars: 250 μm in **(A)**; 1 mm in **(B)**; 500 μm in **(D,F,H)**; Data modified with permission from Smith and Alloway (2014) and Smith et al. (2017). Abbreviations: AGl, agranular lateral cortex; AGm, agranular medial cortex; Cg, cingulate cortex; PrL, prelimbic cortex; dCLA, dorsal claustrum; vCLA, ventral claustrum; ns, neostriatum; ec, external capsule.

Therefore, to compare the anatomical connectivity of rodent homolog of mPFC with the CLA and insular cortex (Figure 5), we made both anterograde and retrograde tracer injections (Fluororuby and Fluorogold, respectively) into Cg cortex (rodent homolog of area 24) and PrL cortex (rodent homolog of area 32; Smith and Alloway, 2014; Smith et al., 2017). Our anterograde tracer injections in Cg cortex revealed connectivity primarily with the contralateral CLA (Figures 5H–K), which in turn innervates only Cg of that hemisphere. Consistent with our rs-fMRI data shown in Figures 3, 4, there was no anatomical connectivity between insula and Cg cortex (Smith and Alloway, 2014; White and Mathur, 2018). Additionally, PrL cortex also sends dense projections to the contralateral CLA and weaker projections to the ipsilateral CLA, with little innervation of the insula in either hemisphere. However, PrL cortex does receive inputs from a large number of neurons in both the CLA and insular cortex of the ipsilateral hemisphere, with very weak projections originating from the contralateral CLA.

**Figure 6:**
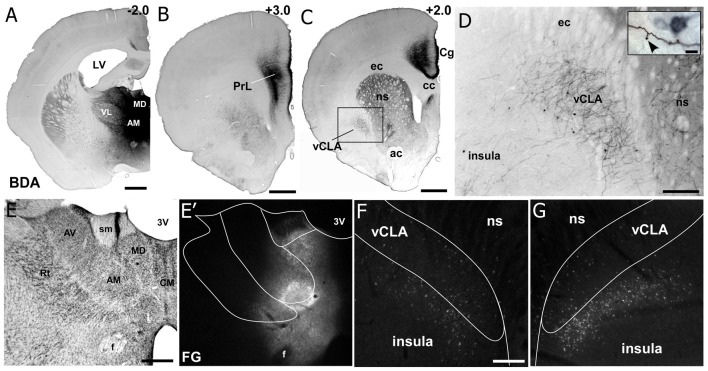
Neuroanatomical tracing from MD thalamus clarifies the connectivity with insular cortex and claustrum. **(A)** Image showing a bi-directional anterograde/retrograde BDA/FG injection into MD thalamus, specifically showing the DAB visualization of the BDA. **(B)** Strong connections are observed with layer 1 as well as layers 5 and 6 of PrL cortex. **(C)** MD thalamus also showed strong connections to Cg cortex, ventral striatum (VS), and CLA. **(D)** Higher magnification of labeling in CLA after MD injection shows mostly terminal labeling, with few retrogradely labeled neurons. Closer inspection of terminals in the inset show a classic “drum-stick” morphology, possibly indicating a “modulator-like” synapse. **(E–G)** Images of the FG labeling from an adjacent section from the injection shown in **(A)**. Not that almost all of the feedback to thalamus originates from insula and not the CLA. Numbers in upper right corner of panels indicate distance from Bregma in millimeter. Scale bars: 1 mm in **(A–C)**; 250 μm in **(D)** and 10 μm in inset. Data modified with permission from Smith et al. (2017). Abbreviations: 3V, third ventricle; LV, lateral ventricle; AM, anteromedial thalamic nucleus; AV, anteroventral thalamic nucleus; VL, ventral lateral nucleus; MD, mediodorsal nucleus; ac, anterior commissure; CM, centromedial thalamic nucleus; cc, corpus callosum; ec, external capsule; ns, neostriatum; Rt, thalamic reticular nucleus; sm, stria medularis; f, fornix; vCLA, ventral claustrum.

Additional tracer injections were made to specify the input-output organization of MD thalamus with mPFC, CLA, and insula. As shown in Figure 6, we made large injections of a mixture of anterograde and retrograde tracers (biotinylated dextran amine with Fluorogold) that targeted MD thalamus (Smith et al., 2017). The resulting anterograde labeling revealed dense projections from MD thalamus to mPFC (including both Cg and PrL cortices) and the CLA, but projections to the insula were largely non-existent (Figures 6B–D). In contrast, the retrograde labeling had the reverse pattern, showing dense labeling in the deep layers of insular cortex, with few cells being found within the CLA (Figure 6G), consistent with previous reports (Mathur et al., 2009).

In addition to the interhemispheric cortico-claustro-cortical circuit linking Cg cortex in each hemisphere, we also observed an interhemispheric cortico-thalamo-cortical loop connecting Cg cortex in each hemisphere *via* MD thalamus (Supplementary Figure S3). We have previously shown a similar interhemispheric loop for the more lateral motor region, agranular medial cortex (AGM, a.k.a. M2), which is involved in rodent whisking (Alloway et al., 2008).

Beyond these classic neuroanatomical tracing approaches, more sophisticated viral tracing from CLA-specific transgenic mice and other approaches have confirmed all of these connections (Chia et al., 2017; Wang et al., 2017; Atlan et al., 2018; Zingg et al., 2018). Furthermore, these studies indicate that there is some reciprocal connectivity between CLA and agranular insular cortex, making it more difficult to interpret differences in functional connectivity between CLA and insula given that these structures appear to have direct connections.

**Figure 7:**
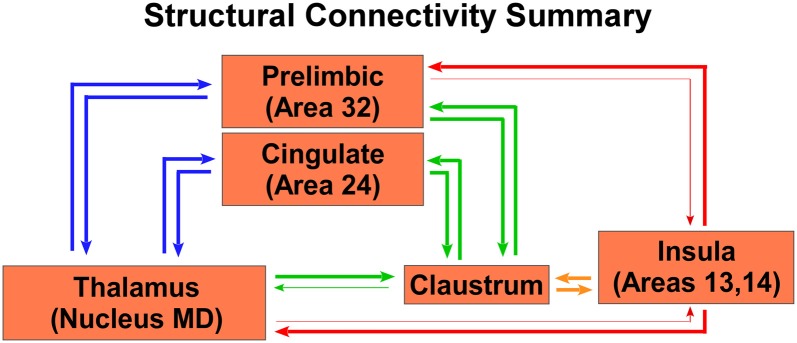
Summary circuit diagram of anatomical connectivity differences between, insula, CLA, PrL, Cg, and MD thalamus. Arrow thickness indicates strength of connection. Parentheticals refer to Brodmann area designations.

A summary of these anatomical results is shown in the circuit diagram in Figure 7, which shows that both the CLA and insula have many similar connections, but three distinct differences. First, though PrL cortex projects primarily to the CLA, it receives inputs from both the CLA and insula. Second, only the CLA has connectivity with Cg cortex. Third, MD thalamus projects strongly to the CLA, but the insula provides the dominant projection back to MD thalamus. Understanding these anatomical differences can provide a lens through which to understand changes in functional connectivity as measured by rs-fMRI.

### An Amygdaloclaustral Circuit to Evaluate Competing Stimuli

Though the function of the CLA remains largely unknown, recent studies indicate that the CLA plays a role in salience detection, attentional processing, and possibly cognition (Remedios et al., 2010, 2014; Goll et al., 2015; Atlan et al., 2018; Jackson et al., 2018), particularly *via* its connections with Cg cortex (Chia et al., 2017; White and Mathur, 2018; White et al., 2018). Most notably, electrophysiology recordings in the CLA of non-human primates lead the investigators to propose that “the claustrum detects the occurrence of novel or salient sensory events” (Remedios et al., 2014). However, it is unknown what brain regions could imbue the CLA with information about the novelty, importance, or relative value of competing stimuli.

**Figure 8:**
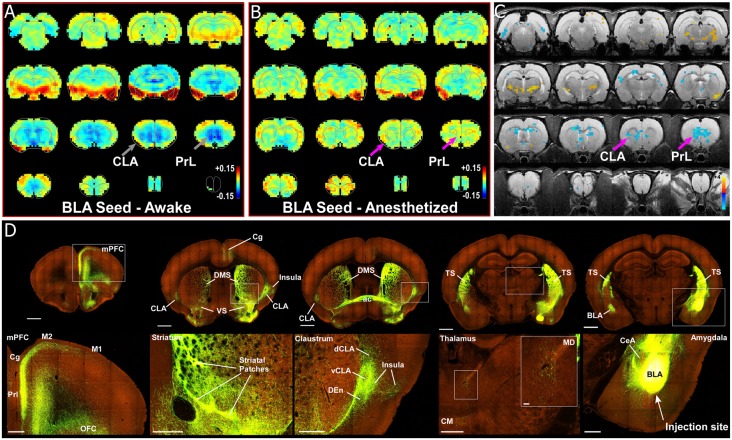
Seed-based analysis and neuroanatomical tracing of basolateral amygdala (BLA) output reveal structural and anti-correlated functional connections with nodes in the SN. **(A)** Unilateral (left hemisphere, images shown in clinical orientation), seed-based analysis of BLA in the awake state. Color plots shown as before with hot colors indicating strong correlations and cool colors indicating anti-correlated blood oxygen level dependent (BOLD) activity. Note the widespread anti-correlation present between BLA and all of mPFC, sensorimotor cortex, dorsal thalamus, and striatum. **(B)** Unilateral, seed-based analysis of BLA in the awake state. Color plots shown as before. **(C)** Statistical map comparing the awake and anesthetized states (voxelwise two-way *t*-test), indicating an increase in correlation strength for functional connections from amygdala to prelimbic cortex (PrL) and claustrum (CLA) in the anesthetized state compared to the awake state (blue area identified by pink arrow). Hot colors show *t*-values for voxels with *p* < 0.05 (false discovery rate), which are significantly weakened in the anesthetized state compared to the awake. Cool colors indicate the reverse, meaning a significant decrease in anti-correlation under anesthesia compared to the awake state. **(D)** Data from the Allen Institute Mouse Connectivity Atlas (Oh et al., 2014) database demonstrate afferent projections from BLA to PrL, Cg, and insular cortices, as well as bilateral projections to striatum and CLA. Image credit: Allen Institute. Scale bars: 1 mm in top row of panel **(D)**; 400 μm in bottom row of panel **(D)**; 40 μm in thalamus inset in bottom row of panel **(D)**.

Some authors suggest that the relative salience of stimuli could be determined by the amygdala, and particularly its inputs to the CLA (Gattass et al., 2014). One unique aspect of our previous rs-fMRI study was the finding that the BLA is negatively correlated (anti-correlated) with infralimbic cortex (Liang et al., 2012a). To further investigate this relationship between the amygdala and nodes of interest within the SN, we re-analyzed our data with respect to the boundaries of the amygdala, as well as performing a unilateral seed-based analysis of the BLA. As seen in Figures 3, 4, unilateral seed-based analyses from the CLA and Cg cortex show that their functional connectivity with the BLA is anti-correlated in the awake state (see blue voxels in the BLA). This is in contrast to the strong correlated BOLD activity observed in other forebrain regions. To verify this anti-correlated BOLD pattern, we performed a unilateral BLA seed analysis (Figures 8A–C). As predicted from our previous analyses, the BLA is anti-correlated with most of the forebrain in the awake state, including areas of mPFC, striatum, septum, thalamus, and CLA.

The precise meaning of anti-correlated functional connectivity is unclear and remains a subject of debate within the rs-fMRI field. It is particularly important to note that negative correlations can result from movement artifacts or data processing methods such as global-signal regression and partial correlation analysis. However, in our studies, we have gone to great lengths to avoid these confounds. First, we do not use global-signal regression as part of our data processing stream. Second, we strictly control for motion artifacts by using stringent motion correction and motion control approaches (full description of our methods can be found in our published research articles: Liang et al., 2011, 2012a, 2015; Smith et al., 2017). As stated above, we previously observed anti-correlated functional connectivity between BLA and infralimbic cortex that was anatomically specific, reproducible, present under various data processing methods, and was observed in a subgroup of animals whose motion levels were the same as anesthetized animals, indicating our findings are functional and not artifactual. Finally, a rs-fMRI study of the BLA in humans has also shown anti-correlated functional connectivity with the caudate and regions of cortex (Roy et al., 2009), similar to our observations in the rodent, thus supporting our findings. Future experiments using electrophysiological recordings in CLA and BLA are required to ascertain the true functional relationship between these regions, which would also be of great value for understanding how to interpret negatively correlated functional rs-fMRI signals in general.

To identify the structural connectivity between the BLA and nodes of interest in the SN, we examined the Allen Mouse Brain Connectivity Atlas for adeno-associated virus (AAV) guided tracing of the BLA (Oh et al., 2014). As shown by a representative example in Figure 8D (Experiment 113144533), injections restricted to the BLA revealed significant projections to a number of cortical (PrL, Cg, and orbitofrontal cortices) and subcortical targets (bilateral CLA and contralateral BLA). As a control, we also inspected similar injections into the adjacent central nucleus of the amygdala (CeA). As shown in Supplementary Figure S4A, and AAV-GFP injection into the CeA (Experiment 146795148) revealed almost no projections to the forebrain, with only weak innervation of ventral striatum (VS), paraventricular thalamus (PV), and the bed nucleus of the stria terminalis (BNST). A summary of the structural connections of the BLA, CeA, and CLA is shown in Supplementary Figure S4B. These data support other recent anatomical articles finding connections between the CLA and BLA (Majak et al., 2002; Atlan et al., 2018; Zingg et al., 2018).

### Claustrum Involvement in a Rodent “Visual Salience Circuit”

**Figure 9:**
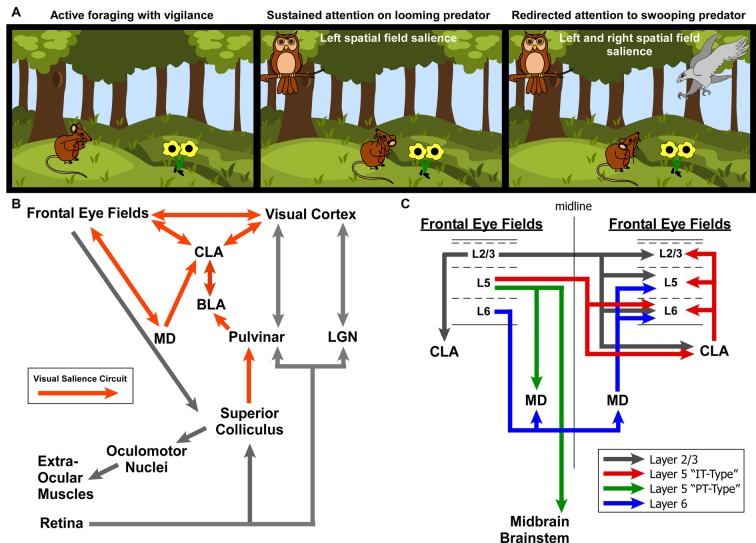
Role for the claustrum in processing visual salience. **(A)** Illustration of ethologically relevant scenario involving rodent vision to orient to predatory salient stimuli while otherwise attempting to actively forage. If predators are in different spatial fields, interhemispheric mechanisms are needed to coordinate attention to these stimuli across disparate visual fields. **(B)** Circuit diagram of the ascending visual processing pathway from the retina and the descending visuomotor pathway for controlling the extra-ocular muscles. Note the proposed “visual salience circuit” emanating from the superior colliculus and relayed to limbic structures (BLA and subsequently CLA) *via* the pulvinar. **(C)** Summary of interhemispheric connections between the rodent frontal eye fields. Summarized from findings from Alloway et al. (2008), Smith and Alloway (2010, 2014) and Smith et al. (2012, 2016, 2017).

To summarize our view of how the CLA might be involved in salience processing, let us consider an ethologically relevant, hypothetical scenario (Figure 9A). Recent behavioral studies in rodent vision have revealed a richer repertoire of functions and versatility than previously appreciated. These studies have shown that the rodent visual system serves a strong role in rodent predation behavior (Hoy et al., 2016), as well as threat detection, particularly regarding over-head, looming stimuli (Wallace et al., 2013; De Franceschi et al., 2016). These overhead visual stimuli likely represent predatory birds, activating a bottom-up visual response from superior colliculus, known to be involved in visual salience (Comoli et al., 2003), which then activates the LP thalamus (pulvinar) and finally the BLA (Uwano et al., 1995; Wei et al., 2015). We now propose that the CLA is the next recipient along this pathway (Figure 9B).

Based on the literature of mammalian oculomotor control (mostly primate studies) and other experiments identifying the frontal eye fields in cingulate cortex of rats (Awh et al., 2006; Smith and Alloway, 2014; Coe and Munoz, 2017), we propose two circuit diagrams for visuomotor control in rodents. In Figure 9B, we propose a “visual salience circuit” (VSC) consisting of both a bottom-up visual processing pathway and a top-down cortical visuomotor control pathway. We propose that the VSC is a combination of limbic structures (including BLA and MD thalamus) that convey information about stimulus value (valence) to the CLA, which in turn coordinates the frontal eye fields and visual cortices to direct attention *via* cortical visuomotor output; ultimately shifting the eyes toward novel, salient stimuli such as unexpected predators (Figure 9A).

When attention needs to be managed across multiple threats in opposing spatial fields (Figure 9C), we hypothesize that the strong bilateral projections from the frontal eye fields to the CLA and MD thalamus support the direct cortico-cortical connections to seamlessly shift attention between both threats. In a recent viral tracing study of the cortico-basal ganglia system, we observed cortical cell-type specificity in the pattern of connectivity with the CLA (Smith et al., 2016). Specifically, we found that the projections to CLA from motor cortex (including Cg cortex, M2, M1) originate from Layer 2/3 and Layer 5 IT-type neurons, but not layer 5 PT-type. This is a crucial anatomical observation that suggests the CLA receives information about motor planning *via* IT-type neurons, but not actual motor output, which is conveyed by the PT-type neurons (Shepherd, 2013). Additionally, Layer 6 of frontal eye fields (and AGm) was not observed to provide inputs to the CLA, confirming our previous retrograde injection in the CLA (Smith and Alloway, 2010). However, Layer 6 was found to have its own interhemispheric circuit with MD thalamus (Supplementary Figure S3), which may support the interhemispheric claustral network to balance attentional loads across spatial fields. These cell-type specific differences may be crucial to understanding how limbic inputs to the CLA are integrated with IT-type information on motor planning from the frontal eye fields to enable coordination between visual cortex and the FEF.

The connectivity between the CLA and Cg cortex is of particular interest with regards to salience processing. The human SN is defined as coupling between the dACC and a region that appears to encompass the CLA. In addition to our seed-based analysis that corroborates this functional connection in rodents, we have demonstrated anatomical and functional connections from the CLA to Cg cortex. The CLA to Cg cortex circuit seems well-suited to guide salience processing owing to the limbic inputs received by the CLA from the BLA, which are relayed to Cg cortex: a motor output region shown to guide eye movements (Smith and Alloway, 2014). This circuit organization suggests a functional role for the CLA in attention as a “limbic-to-motor” interface. A recent study corroborates the importance of the CLA-Cg connection in salience processing by demonstrating that Cg cortex imbues the CLA with “top-down” expectation signals related to task-relevant sensory information that likely underlies attentional mechanisms (White et al., 2018). Another recent study also found behavioral evidence that the CLA provides resilience to distraction (Atlan et al., 2018), which is another mechanism of attention. We, therefore, believe the CLA-Cg circuit plays a prominent role in the SN and suggest that future studies investigating the function of the CLA focus on its potential role in salience processing. Such circuitry could underlie the classic “cocktail party problem” of attending to a specific stimulus amongst competing noise. Other classic experiments in attention are ripe for testing the involvement of the CLA.

### Clinical Implications

Major connectivity networks such as the DMN and SN can have aberrant connectivity patterns that are thought to underlie a host of neurological and psychiatric disorders (Menon, 2011; Uddin, 2015). For example, as shown in Figure 1B, a strong correlation exists between a subject’s level of anxiety and the connectional strength of the SN, suggesting a role in anxiety disorders and pathological states of hyper vigilance (Seeley et al., 2007; Geng et al., 2016). A variety of other psychiatric disorders are also correlated with the insula and salience dysfunction, including depression (Menon, 2011), psychosis with auditory delusions (Mallikarjun et al., 2018), post-traumatic stress disorder (Brown et al., 2014), affective disorders (Menon and Uddin, 2010), as well as autism, schizophrenia, and dementia (Calaitzakis et al., 2009; Uddin, 2015).

Based on our recent studies, we propose that the CLA plays a role in SN-related neuropsychiatric disorders (Kalaitzakis et al., 2009; Cascella et al., 2011; Smith and Alloway, 2014; Wegiel et al., 2014; Patru and Reser, 2015; Bernstein et al., 2016; Smith et al., 2017). A recent fMRI study of Parkinson’s disease patients has found significant decreases in the functional connectivity of the CLA compared to age-matched control patients, especially “with areas mainly involved in visuomotor and attentional systems” (Arrigo et al., 2018). Due to their similar anatomical and functional connections with Cg cortex and the BLA, both the insula and CLA could play a role in regulating levels of vigilance through the SN, which underlie a host of affective disorders.

Future neuroimaging and optogenetic behavioral studies are necessary to demonstrate what role, if any, the CLA plays in emotional processing, particularly with regard to anxiety. Nevertheless, this endeavor represents a new frontier for the treatment of affective neuropsychiatric disorders using deep brain stimulation or drugs that target the CLA. In fact, kappa opioid receptors, which are abundantly expressed in the CLA, may serve a therapeutic role in treating depression (Stiefel et al., 2014). Future research on the CLA will no doubt lead to fundamental breakthroughs in our understanding of emotional processing in health and disease.

## Conclusion

Our results demonstrate a role for the CLA in both the DMN and the SN. Furthermore, these findings expand the view of the CLA from being only a sensorimotor structure (Smith and Alloway, 2014), and suggest it has a role in emotional and salience processes, owing to its anatomical and functional connectivity with limbic and affective brain regions (Figure 9). Together with recent behavioral studies, we propose the CLA serves as a limbic-sensorimotor interface, facilitating salience-guided orienting during sensory exploration.

## Ethics Statement

This study was carried out in accordance with the guidelines and approval of the Institutional Review Board at the University of Massachusetts and Penn State University.

## Author Contributions

JS and GW wrote the first draft of the manuscript, constructed figures, and performed anatomy experiments and analysis. JS, GW, NZ and KA made significant revisions to multiple drafts of the manuscript. ZL, YL and NZ performed rs-fMRI experiments and analysis. NZ and KA obtained funding and supervised all data collection and analysis. All authors approve this submission and take responsibility for all information contained in this article.

## Conflict of Interest Statement

The authors declare that the research was conducted in the absence of any commercial or financial relationships that could be construed as a potential conflict of interest.

## Supplementary Material

FIGURE S1 Claustrum and insula ROIs overlaid on DMN correlation maps. Top row shows the correlation maps from Figure 2 from a unilateral mPFC seed analysis in the awake (left side) and anesthetized (right) conditions, with the voxels from our CLA (black) and insula (magenta) seed analyses (from Figure 4) superimposed. Bottom row is organized in the same manner for our MD thalamus seed analysis from Figure 2.

FIGURE S2 Claustrum and insula ROIs overlaid on SN correlation maps. Top row shows the correlation maps from Figure 3 from a unilateral insula seed analysis in the awake (left side) and anesthetized (right) conditions, with the voxels from our CLA (black) and insula (magenta) seed analyses (from Figure 4) superimposed. Bottom row is organized in the same manner for our Cg cortex seed analysis from Figure 3.

FIGURE S3 Interhemispheric cortico-thalamo-cortical loop between mPFC and Cg *via* MD thalamus. **(A)** Thalamic labeling from the anterograde and retrograde injections in PrL cortex shown in Figures 5A–C. Note the anterogradely labeled terminals in the right hemisphere MD thalamus (contralateral to the FR injection site), which are intermingled with retrograde neurons from the FG injection in the right hemisphere PrL. **(B–E)** Thalamic labeling from the BDA injection into Cg cortex shown in Figure 5I. Note the terminal labeling in the contralateral MD. Data modified with permission from Smith and Alloway (2014) and Smith et al. (2017). Scale bars: 500 μm in left image of panel **(A)**; 50 μm in right image of panel **(A)**; 250 μm in **(B,D)**; 100 μm in **(C)**. Abbreviations: 3V, third ventricle; AM, anteromedial thalamic nucleus; AV, anteroventral thalamic nucleus; VL, ventral lateral nucleus; VM, ventral medial nucleus; MD, mediodorsal nucleus; Re, reuniens; IAM, interanteromedial nucleus; ac, anterior commissure; CM, centromedial thalamic nucleus; cc, corpus callosum; ec, external capsule; ns, neostriatum; Rt, thalamic reticular nucleus; sm, stria medularis; f, fornix; vCLA, ventral claustrum.

FIGURE S4 Anatomical connectivity of central amygdala. **(A)** Projections from central nucleus of the amygdala (CeA) by contrast only weakly target the ventral striatum (VS) and bed nucleus of the stria terminalis (BNST) in the forebrain. **(B)** Summary diagram of afferents from the BLA, CeA, CLA and BNST. Scale bars: 1 mm in top row of panel **(A)**; 400 μm in bottom row of panel **(A)**; 40 μm in thalamus inset in bottom row of panel **(A)**. Data from the Allen Institute Mouse Connectivity Atlas (Oh et al., 2014). Image credit: Allen Institute.
