# Supplementary figures and images for: A Role for the Claustrum in Salience Processing?
Source: Front Neuroanat. 2019 Jun 19;13:64. doi: 10.3389/fnana.2019.00064 (PMC6594418; doi:10.3389/fnana.2019.00064)

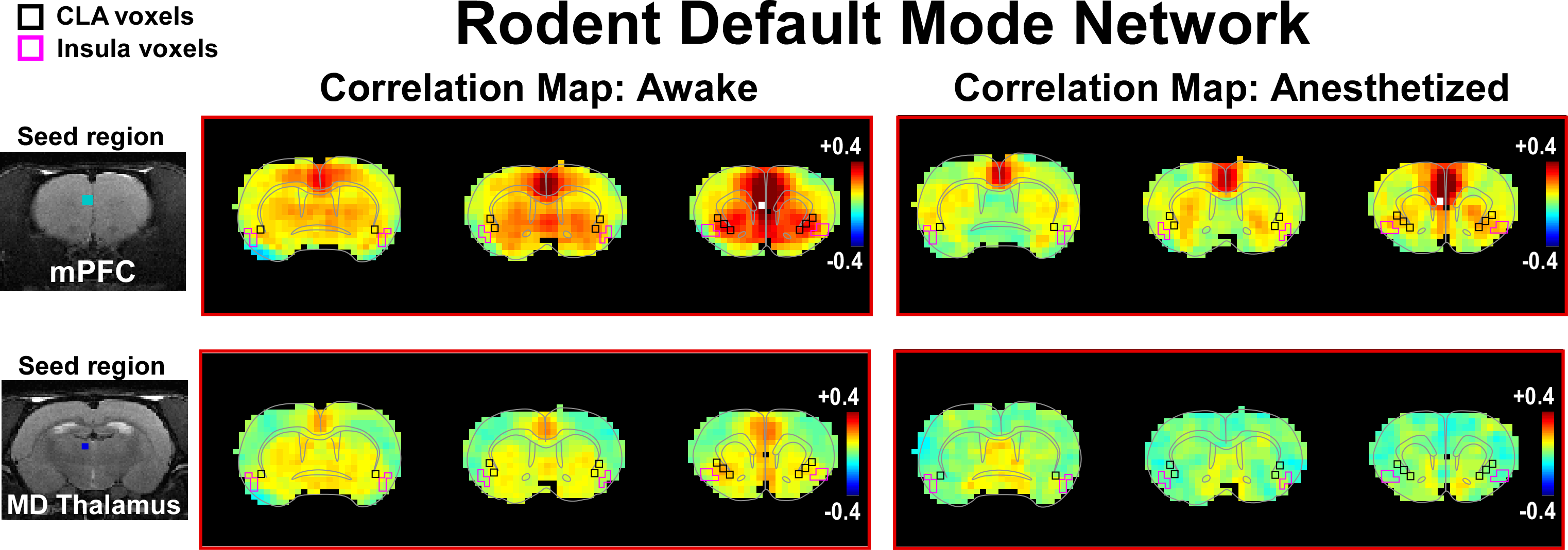

Supplement: FIGURE S1 — Claustrum and insula ROIs overlaid on DMN correlation maps. Top row shows the correlation maps from Figure 2 from a unilateral mPFC seed analysis in the awake (left side) and anesthetized (right) conditions, with the voxels from our CLA (black) and insula (magenta) seed analyses (from Figure 4) superimposed. Bottom row is organized in the same manner for our MD thalamus seed analysis from Figure 2. [file Image_1.TIF]

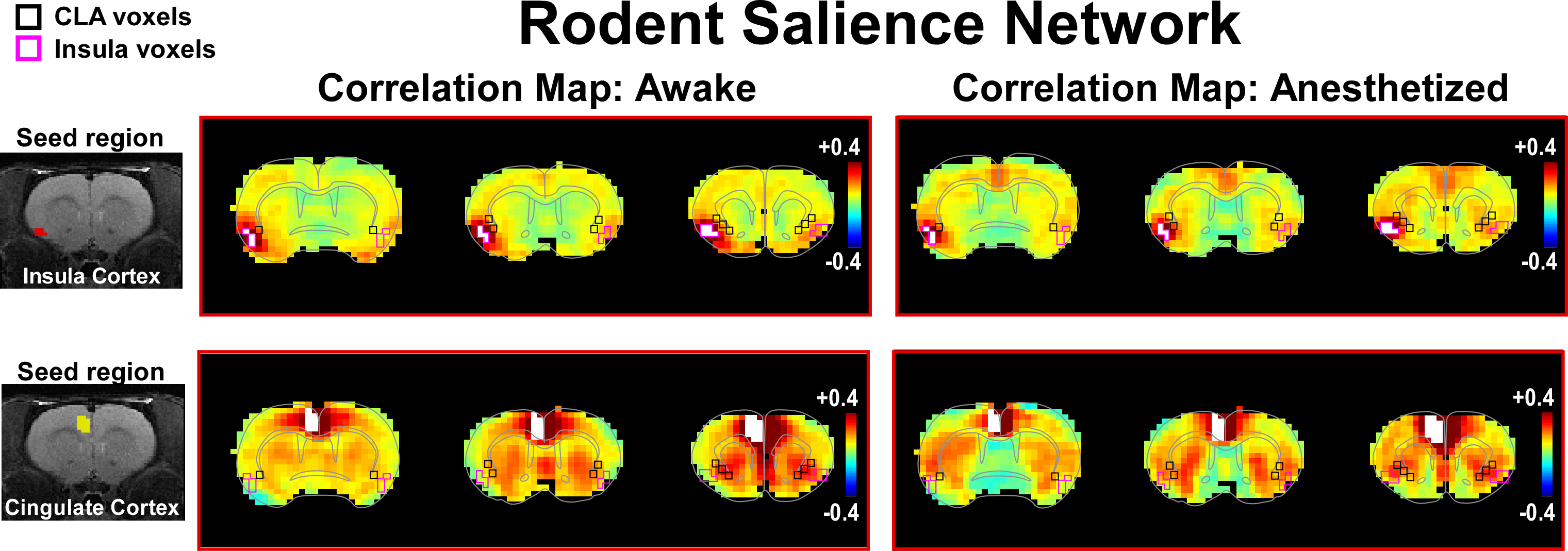

Supplement: FIGURE S2 — Claustrum and insula ROIs overlaid on SN correlation maps. Top row shows the correlation maps from Figure 3 from a unilateral insula seed analysis in the awake (left side) and anesthetized (right) conditions, with the voxels from our CLA (black) and insula (magenta) seed analyses (from Figure 4) superimposed. Bottom row is organized in the same manner for our Cg cortex seed analysis from Figure 3. [file Image_2.TIF]

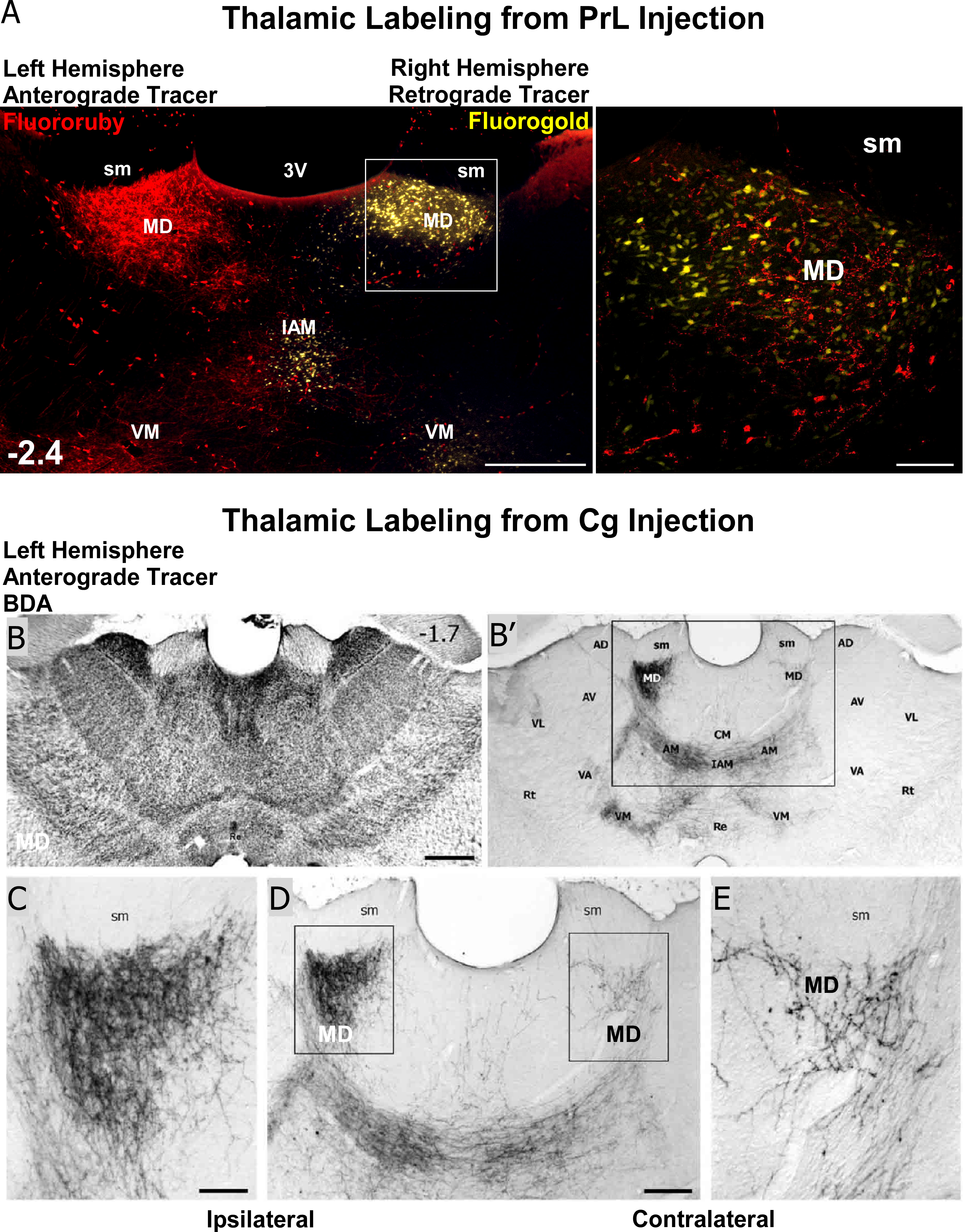

Supplement: FIGURE S3 — Interhemispheric cortico-thalamo-cortical loop between mPFC and Cg via MD thalamus. (A) Thalamic labeling from the anterograde and retrograde injections in PrL cortex shown in Figures 5A–C. Note the anterogradely labeled terminals in the right hemisphere MD thalamus (contralateral to the FR injection site), which are intermingled with retrograde neurons from the FG injection in the right hemisphere PrL. (B–E) Thalamic labeling from the BDA injection into Cg cortex shown in Figure 5I. Note the terminal labeling in the contralateral MD. Data modified with permission from Smith and Alloway (2014) and Smith et al. (2017). Scale bars: 500 μm in left image of panel (A); 50 μm in right image of panel (A); 250 μm in (B,D); 100 μm in (C). Abbreviations: 3V, third ventricle; AM, anteromedial thalamic nucleus; AV, anteroventral thalamic nucleus; VL, ventral lateral nucleus; VM, ventral medial nucleus; MD, mediodorsal nucleus; Re, reuniens; IAM, interanteromedial nucleus; ac, anterior commissure; CM, centromedial thalamic nucleus; cc, corpus callosum; ec, external capsule; ns, neostriatum; Rt, thalamic reticular nucleus; sm, stria medularis; f, fornix; vCLA, ventral claustrum. [file Image_3.TIF]

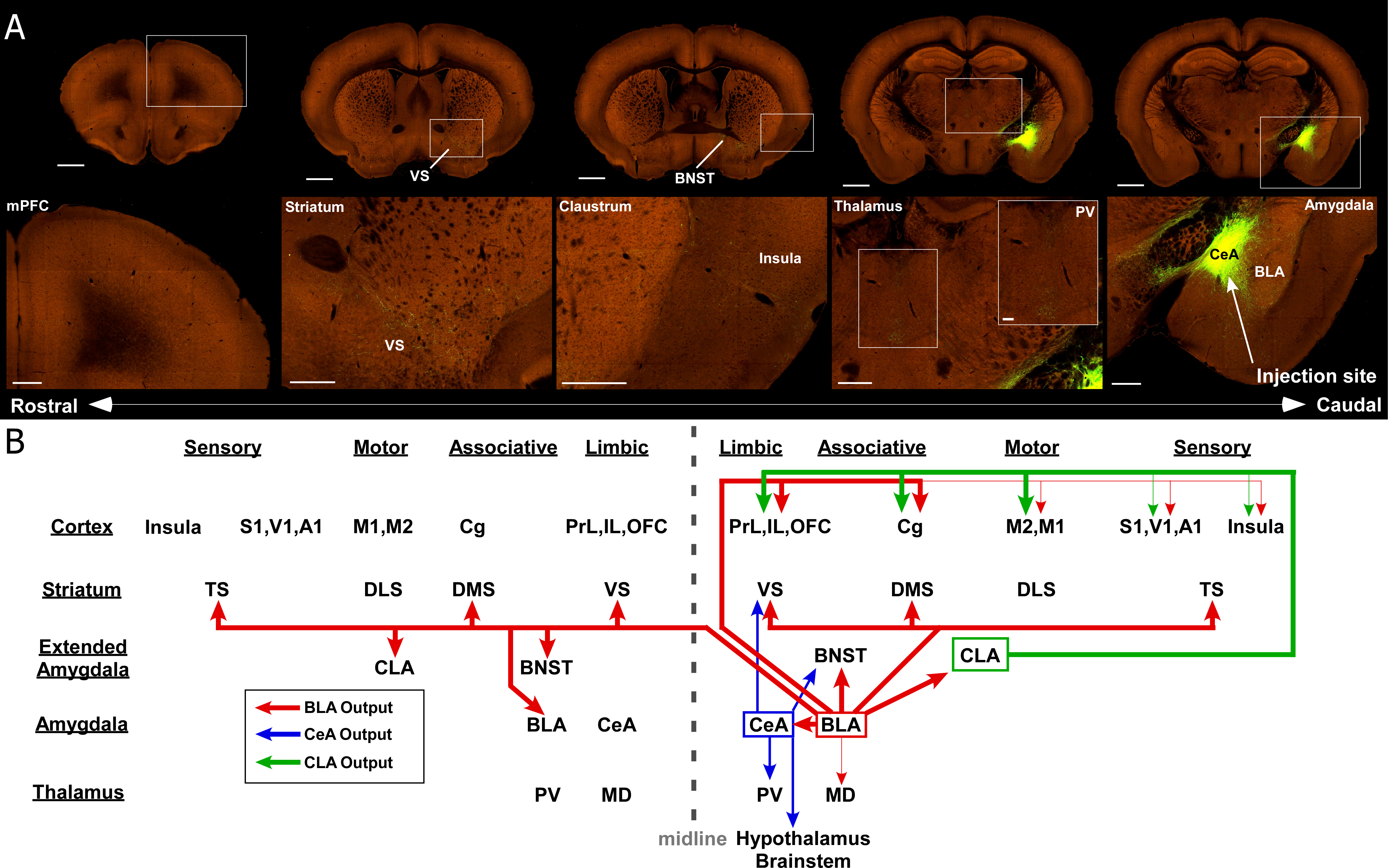

Supplement: FIGURE S4 — Anatomical connectivity of central amygdala. (A) Projections from central nucleus of the amygdala (CeA) by contrast only weakly target the ventral striatum (VS) and bed nucleus of the stria terminalis (BNST) in the forebrain. (B) Summary diagram of afferents from the BLA, CeA, CLA and BNST. Scale bars: 1 mm in top row of panel (A); 400 μm in bottom row of panel (A); 40 μm in thalamus inset in bottom row of panel (A). Data from the Allen Institute Mouse Connectivity Atlas (Oh et al., 2014). Image credit: Allen Institute. [file Image_4.TIF]
